# Respiratory Syncytial Virus: The Urgent Need for Innovative Preventive Strategies

**DOI:** 10.3390/pediatric16030057

**Published:** 2024-08-08

**Authors:** Fabrizio Virgili, Fabio Midulla, Fernando Maria de Benedictis

**Affiliations:** 1 Department of Maternal Infantile and Urological Sciences, Sapienza University of Rome, 00185 Rome, Italy; fabrizio.virgili@uniroma1.it (F.V.); midulla@uniroma1.it (F.M.); 2 Salesi Children’s Hospital Foundation, 60123 Ancona, Italy

Respiratory Syncytial Virus (RSV) is a medium-sized enveloped Pneumovirus belonging to the Paramyxoviridae family. It is equipped with a single negative-sense RNA chain encoding for 11 proteins, among which the F protein (which facilitates epithelial cell binding) and the G protein (which allows syncytia formation), are both immunogenic. There are more than 50 different RSV genotypes related the G protein, leading to variability which contributes to viral pathogenicity and complicates the development of an effective vaccine [1,2].

RSV transmission is airborne, occurring through direct contact with respiratory secretions from infected individuals who might experience only mild respiratory symptoms like rhinitis [3,4]. The virus is ubiquitous, and it is estimated that virtually all children contract the infection at least once within the first two years of life. The epidemiology of RSV infections varies with meteorological conditions: in the northern hemisphere, transmission rates increase from November to March; in the southern hemisphere, the epidemic peak takes place in May–July. The Public Health measures introduced during the COVID-19 pandemic resulted in a dramatic drop in viral circulation; nonetheless, the rebound effect following the relaxation of these disease management efforts has been characterized by a heavier disease burden, with infection rates peaking outside the conventional epidemic season and age range [5,6,7]. RSV constitutes the main causative agent of bronchiolitis, a seasonal illness representing the most prevalent acute inflammatory infection of the lower respiratory tract during the first year of life. 

Bronchiolitis is the primary cause of pediatric medical admissions in developed countries, with approximately 33 million new cases annually worldwide, 10% of which necessitate hospital admission [8,9,10]. A significant socioeconomic burden (almost EUR 5 billion per year) is also linked to the complications that RSV infections entail, as increasing evidence points out a correlation with the subsequent development of recurrent wheezing, asthma, and impaired lung function. Nonetheless, it remains uncertain whether this viral infection predisposes certain individuals to asthma through a TH2 response, or if the incidence of RSV infections is higher in subjects that are already vulnerable [11,12,13,14].

The diagnosis of bronchiolitis is clinical. Blood gas analysis, chest X-ray, and lung ultrasound are not usually required, but may be useful in severe cases or those with a degree of uncertainty. Pathogen identification is generally not recommended, except for the purposes of epidemiological research, which confirms that Realtime Polymerase Chain Reaction performed on samples from the upper airways is the most sensitive test [15,16,17,18,19].

Despite general agreement on the overall management of bronchiolitis, significant inconsistencies among international recommendations reflect the paucity of relevant evidence in some fields of commonly accepted practice. International guidelines highlight that the management of bronchiolitis is supportive rather than causal or interventional, focusing on respiratory and metabolic stabilization.An adequate fluid intake should be ensured by administering small but frequent quantities of oral liquids. If oral feeding is not possible, fluids may be provided via a nasogastric tube or intravenous rehydration [20]. Nasal washing before meals and as needed may improve upper airway obstruction and reduce respiratory effort, encouraging feeding [21]. In the case of desaturation, low-flow oxygen therapy can be administered through nasal cannulas or a face mask. Heated Humidified High-Flow Nasal Cannula Oxygen should be considered if high cardiac and/or respiratory rates persist or if SatO2 levels remain low despite low-flow oxygen supplementation [22,23,24,25]. According to current indications, Continuous Positive Airway Pressure ventilation through the nose proves to be an alternative or—in some cases—a rescue therapy in the event of HHHFNCO failure [23,25,26,27]. To date, there is limited or inconsistent evidence supporting alternative therapeutic options (e.g., nebulized hypertonic saline, ribavirin, ipratropium, epinephrine, and systemic or inhaled corticosteroids), which, despite being widely used, are not recommended. Recent guidelines advise against the use of β2-agonists, as meta-analyses show no benefit in terms of saturation improvement, hospital admission rates, or length of stay. Nevertheless, in moderate to severe cases, a trial of aerosolized albuterol may identify patients who could potentially benefit from its use [28,29,30,31,32,33].

Given the limited possibilities in terms of causal therapy, prevention strategies seem pivotal in reducing the incidence of bronchiolitis. Several factors support the development of vaccines against RSV: the early onset of infection and the resulting socio-economic burden; the neutralizing ability of RSV-specific antibodies; and the low mutation rate and antigenic consistency of the F protein. In 1960s, after recognizing RSV as a potential cause of respiratory illness in infants, the scientific community promptly mobilized to develop immunization strategies. However, immunization with a formalin-inactivated whole-virus vaccine led to more severe disease after subsequent natural RSV infection in infants [34]. Consequently, pharmacological research has focused on passive immunization technologies, specifically monoclonal antibodies [35,36,37]. 

Palivizumab, which is administered monthly (15 mg/kg intramuscularly) during the RSV epidemic season, is a monoclonal antibody against the RSV F protein. Due to its cost-effectiveness, it is recommended for high-risk infants: neonates born with a gestational age under 29 weeks; infants born with a gestational age under 32 weeks and suffering from chronic lung disease; infants with hemodynamically significant heart disease; and children below 2 years of age undergoing post-cardiac transplant prophylaxis. Systematic reviews have proven the adequate safety profile of Palivizumab and its efficacy in reducing hospitalizations, though it has no significant impact on mortality or adverse events [38,39].

Nirsevimab is a recently approved recombinant monoclonal antibody targeting a highly conserved epitope on the RSV F protein (site Ø of the prefusion protein), thereby blocking viral entry. It seems to provide multiple advantages: effectiveness after a single dose (50 mg < 5 kg; 100 mg ≥ 5 kg); prolonged half-life (5 months) due to triple amino acid substitutions in the fragment crystallizable region of the antibody; and strengthened immunization, since maintaining the prefusion conformation of the F protein prefusion enhances the production of neutralizing antibodies [40]. Trials have shown that a single injection before the epidemic season can prevent RSV-associated lower respiratory tract infections in both preterm and term infants [41,42,43]. Recent data have emerged from an analysis of the effectiveness of Nirsevimab in a real-world setting during the first epidemic season after its approval (October 2023–February 2024); it was confirmed that the medication successfully reduced RSV-related hospitalization rates, with an efficacy ranging from 70% to 90% across different countries [44,45].

A further promising candidate is Clesrovimab, an extended half-life monoclonal antibody currently in phase III trials. Model-based analyses suggest with reasonable probability that a single 75 mg dose could ensure substantial protection (75% of efficacy) for up to five months in term infants [46]. Early-stage clinical trials are evaluating another potential long-acting fully human monoclonal antibody named TNM001 (Trinomab) [47]. Nonetheless, the affordability of new monoclonal antibodies remains a hindrance to their widespread use, particularly in resource-limited settings. In light of the limited viral resistance developed over the years, Palivizumab will remain a reference molecule in the market to cover the temporary and spatial gap in the global distribution of extended-life antibodies and new vaccines [35].

Although active immunization for pediatric use is still currently unavailable, two bivalent subunit vaccines (Arexvy^®^ and Abrysvo^®^) targeting the prefusion protein (RSVpreF) were introduced to global market in 2023, proving to be moderately to highly effective in preventing RSV-related lower respiratory tract infections in adults > 60 years old [48,49].

Maternal active immunization is a compelling option for protecting infants under 6 months old through the delivery of transplacental antibodies. Recent phase III placebo-controlled trial results for a bivalent RSV prefusion vaccine (Abrysvo^®^) administered to women 32–36 weeks pregnant have been encouraging, demonstrating significant efficacy in protecting infants from severe RSV infections. Abrysvo^®^ exhibited an 82% effectiveness in preventing medically attended severe LRTI in the initial 90 days of life and 69% effectiveness in the first 6 months, with no safety concerns observed in vaccinated mothers or newborns. However, there was no statistically significant difference in medically attended RSV-associated lower respiratory tract infections between the vaccine and placebo groups [50,51].

With the availability of both infant immunoprophylaxis and maternal vaccines, it is intriguing to envision how these preventive strategies will synergistically coexist and interact. Monoclonal antibodies remain useful due to their efficacy in protecting both premature and full-term infants, their protracted protection, their adaptability to variable RSV seasonality, and their applicability in settings and instances where maternal immunization has not been administered. Conversely, maternal vaccines provide comprehensive protection and may represent a contingency in scenarios of viral resistance to antibodies and in the face of the prohibitive production costs of monoclonal antibodies

Current research is also investigating alternative vaccine strategies, including live-attenuated, vector-based, and nucleic acid-based vaccines, though no trial on the pediatric population has progressed to phase III yet [52]. According to the results of a double-blind, placebo-controlled trial, a single intranasal dose of a c-DNA-derived live-attenuated vaccine (RSV/6120/ΔNS2/1030s) proved immunogenic and genetically stable in RSV-seronegative infants, with only minor side effects (rhinorrhea) [53].

An intriguing quandary arises regarding the potential impact of primary prevention on the microbiological epidemiology of respiratory infections. While Palivizumab has been shown to decrease RSV-related LTRI, recent findings indicate no significant differences in the overall incidence of bronchiolitis, suggesting that a shift in the microbial etiology of this disease seems inevitable. Therefore, continuous surveillance and adaptive strategies will be crucial in managing the evolving landscape of respiratory infections [54].

RSV remains a significant global health challenge due to its high prevalence and associated complications, particularly in infants and the elderly.The virus’s ubiquity and the severe burden it places on healthcare systems underscore the importance of multifaceted preventive strategies. While advances in the production of monoclonal antibodies offer substantial protection, their high costs limit their widespread use, particularly in resource-limited settings. The recent development of subunit vaccines for adults and promising results from maternal immunization trials highlight the potential for comprehensive prevention strategies. The ongoing research exploring innovative vaccine platforms, including vector- and nucleic acid-based vaccines, suggests a future with more diverse and effective immunization options. The synergistic development and implementation of these strategies, combined with ongoing surveillance, could significantly reduce the global burden of RSV, improving outcomes for the most vulnerable populations.

## Data Availability

Data sharing is not applicable to this article as no datasets were generated or analyzed during the current study.
